# A Probabilistic Approach to Estimating Allowed SNR Values for Automotive LiDARs in “Smart Cities” under Various External Influences

**DOI:** 10.3390/s22020609

**Published:** 2022-01-13

**Authors:** Roman Meshcheryakov, Andrey Iskhakov, Mark Mamchenko, Maria Romanova, Saygid Uvaysov, Yedilkhan Amirgaliyev, Konrad Gromaszek

**Affiliations:** 1 V. A. Trapeznikov Institute of Control Sciences of Russian Academy of Sciences, 117997 Moscow, Russia; iskhakovandrey@gmail.com (A.I.); markmamcha@gmail.com (M.M.); metelisama@gmail.com (M.R.); 2 Department of Design and Production of Radio-Electronic Means, Institute of Radio Engineering and Telecommunication Systems, MIREA—Russian Technological University, 119454 Moscow, Russia; uvaysov@yandex.ru; 3 Institute of Information and Computational Technologies CS MES RK, Almaty 050010, Kazakhstan; amir_ed@mail.ru; 4 Department of Electronics and Information Technology, Faculty of Electrical Engineering and Computer Science, Lublin University of Technology, ul. Nadbystrzycka 38d, 20-618 Lublin, Poland; k.gromaszek@pollub.pl

**Keywords:** unmanned vehicles, light detection and ranging, LiDAR, signal-to-noise ratio, false alarm, laser, threshold value, probability characteristics

## Abstract

The paper proposes an approach to assessing the allowed signal-to-noise ratio (SNR) for light detection and ranging (LiDAR) of unmanned autonomous vehicles based on the predetermined probability of false alarms under various intentional and unintentional influencing factors. The focus of this study is on the relevant issue of the safe use of LiDAR data and measurement systems within the “smart city” infrastructure. The research team analyzed and systematized various external impacts on the LiDAR systems, as well as the state-of-the-art approaches to improving their security and resilience. It has been established that the current works on the analysis of external influences on the LiDARs and methods for their mitigation focus mainly on physical (hardware) approaches (proposing most often other types of modulation and optical signal frequencies), and less often software approaches, through the use of additional anomaly detection techniques and data integrity verification systems, as well as improving the efficiency of data filtering in the cloud point. In addition, the sources analyzed in this paper do not offer methodological support for the design of the LiDAR in the very early stages of their creation, taking into account a priori assessment of the allowed SNR threshold and probability of detecting a reflected pulse and the requirements to minimize the probability of “missing” an object when scanning with no a priori assessments of the detection probability characteristics of the LiDAR. The authors propose a synthetic approach as a mathematical tool for designing a resilient LiDAR system. The approach is based on the physics of infrared radiation, the Bayesian theory, and the Neyman–Pearson criterion. It features the use of a predetermined threshold for false alarms, the probability of interference in the analytics, and the characteristics of the LiDAR’s receivers. The result is the analytical solution to the problem of calculating the allowed SNR while stabilizing the level of “false alarms” in terms of background noise caused by a given type of interference. The work presents modelling results for the “false alarm” probability values depending on the selected optimality criterion. The efficiency of the proposed approach has been proven by the simulation results of the received optical power of the LiDAR’s signal based on the calculated SNR threshold and noise values.

## 1. Introduction

Almost all modern autonomous vehicles (AVs) are equipped with light detection and ranging (LiDAR) devices. This technology allows to obtain and process data on the remote objects using active optical (laser) systems. These sensors are physically based on the use of light absorption and scattering phenomena in optically transparent (and semi-transparent) media. However, these sensors are among those most exposed to external influences in self-driving systems [1,2]. Incorrect input from LiDAR can lead to the incorrect behavior of the AVs on the roads, traffic disruption, and accidents, posing risks to humans’ health and life. The “smart city” concept implies that a large number of unmanned vehicles will be driving simultaneously, and this poses the major problem of the usage of the LiDARs, the proven influence of the LiDARs on each other, i.e., the mutual interference of the signals from several LiDARs. In addition, a number of problems related to the use of LiDARs in various weather conditions, times of day and year remains unresolved. In particular, LiDAR’s functioning can be influenced by the sunlight, low temperatures, and harsh weather conditions, e.g., rain, snow, fog, wind, etc., resulting in their performance degradation [3,4,5].

Furthermore, the LiDARs may be intentionally affected by attackers with the aim of both altering the LiDAR’s data and completely disabling it. The attacker’s range of possible actions is wide and includes both common (changing the LiDAR’s point cloud, injecting fake echoes in the received signal, and “blinding” the sensor with a laser beam), and advanced (manipulation of raw point cloud data and fusion processes, conducting side-channel attacks, crafting adversarial objects and using adversarial machine learning techniques to deceive the LiDAR’s detection system, using malware to infect the LiDAR’s firmware) types of attack. The situation is compounded by the presence of detailed descriptions of most of the above-mentioned attacks, and the availability and low cost of the basic equipment required to conduct them. Such malicious acts can lead to serious traffic accidents if the attack is successful.

Thus, it is obvious that the LiDAR’s operation is accompanied by multiple permanent disturbances (noise), and this problem is one of the major factors hindering the widespread use of automotive LiDARs on the roads of the “smart cities”. This also supports the relevance of the problem of useful signal detection in the context of background noise in LiDAR systems.

To reduce the influence of interference, noise, and other above-described negative factors, one can apply techniques that can be broadly divided into design solutions (during the stage of development) and methodological ones (using the mathematical tool) can be applied. The design of an optoelectronic system involves all basic physical parameters for pulse transmission-wavelength, amplitude, phase, polarization state, etc. But there is no independent physical parameter left during the noise extraction and calculation of the SNR. These methods require additional optical equipment with high-speed modulation capability [6], increasing the cost of the LiDAR and the delay of object detection. In contrast to technological approaches, mathematical methods are less expensive and easier to implement. The complexity of determining the value of the SNR can lead to additional errors, since the input data have different probability distributions. Even if the SNR values at the output of the receiving system are equal, the probabilistic detection characteristics (probability of correct detection and false alarm) can be different. In such cases, it is correct to perform a Neyman–Pearson criterion estimation of the detection characteristics.

The purpose of this study is to analyze the probability characteristics for the power flow obtained theoretically and calculate the Neyman–Pearson criterion. Based on this criterion, it is possible to develop an analytical algorithm for estimating the SNR threshold values for stabilizing the false alarms level with possible interferences of multiple reflected pulses. To stabilize the false alarms level, it is necessary to define a distribution of power statistics of both input and output pulses according to the formulated problem on two hypotheses. An additional feature of the problem to be solved is the non-Gaussian law of distribution of statistical observations of pulses power. To achieve that objective, it is important to conduct a comprehensive review and systematization of existing intentional and unintended impacts on the LiDARs, as well as state-of-the-art methods of increasing their resilience.

The article is organized as follows. Section 2 presents theoretical aspects of the study of diffuse interference, including the physical basis of laser radiation and the reflectivity of the object, as well as methods for calculating the distance from the LiDAR to an object. Section 3 contains the analysis of work related to threats and vulnerabilities of LiDAR sensor systems. The section provides an overview of the main cyber-attacks on both sensor and model levels of LiDARs of the AVs. A systematization of the examined attacks is given, and various approaches to LiDAR attacks classification used in the analyzed scientific literature are considered. Section 4 gives the classification of unintentional adverse impacts that may be caused by either direct or back-scattered interference from one or more LiDAR located near the target one, as well as by environmental effects (sunlight radiation, snow, rain, wind, clouds, air humidity, wet road surface, etc.). Section 5 provides an overview of known recommendations and methods to counter or mitigate relevant attacks on the automotive LiDARs in the “smart city” environment, as well as common techniques to improve the resilience of the LiDAR systems to unintended external impacts. Section 6 proposes an approach to the detection of the echo signal against a background of noise, as well as calculating the probability of identifying the “false alarm” event of the target automotive LiDAR under the influence of both direct and backscattered interference. The simulation results of the “false alarm” probability are presented depending on the selected optimality criterion. Section 7 presents the consolidation of the paper’s results.

## 2. Theoretical Aspects of the Study of Diffuse Interference

### 2.1. Physical Basis of Laser Radiation

It is known that laser radiation has a number of properties such as monochromaticity, coherence, and polarization, so the operation of semiconductor optical devices may differ from that of optical devices with broadband radiation. Due to monochromaticity, accurate energy calculations require data on spectral transmittance values rather than integral ones. This property of laser radiation also increases the criticality of semiconductor optics functioning to external conditions: pressure, humidity, temperature, etc. Coherence of laser radiation also affects the operation of optical systems. It is mainly related to the possibility of undesirable interference effects. Polarization of laser radiation leads to the fact that the reflection coefficients from the interface of two media depend not only on the angle of incidence, but also on the polarization state. Recall that there are incidence angles at which the reflection coefficient at a certain polarization state is zero (Brewster’s angle). If the polarization state of the laser output should be kept also at the output of the optical system, it should be calculated in such a way that the incident angles do not exceed the critical values at which the polarization state does not change (up to 10°).

### 2.2. Methods of Forming a Multibeam Structure

Depending on how the multibeam laser structure is formed, LiDARs can be divided into the following types: LiDARs with a line of lasers, LiDARs using matrix lasers, and LiDARs with diffractive optical elements.

#### 2.2.1. Lidars with a Line of Lasers

In these types of LiDAR, the scanning is in one direction, along the *x*-axis only. In order to provide spatial scanning in orthogonal direction by a set of beams, it is necessary to arrange the lasers in a vertical line. In this case, lasers, the transmitting optical system, the receiving optical system, and the receivers are located in the body installed on the rotation axis.

As the body rotates, parallel beams of radiation are simultaneously sent into space, and the beams reflected from objects arrive at the receivers corresponding to each laser beam. The distance to the reflecting surface of the object can be determined in two ways: either by the time-of-flight method or by the appropriate phase shift of the pulses. The angular azimuthal coordinate is determined by the rotation angle of the platform, while the elevation is determined by the angular position of the respective laser. This multibeam LiDAR arrangement is most commonly used in automotive active safety systems and unmanned vehicles. For example, Velodyne is developing multibeam scanning LiDARs in the HDL-64 or HDL-128 types to be installed in ADAS and autopilot systems in order to obtain all-round panoramas [7].

#### 2.2.2. Lidars Using Matrix Lasers

A more promising technology which is cheaper, faster, offers higher resolution, and will eventually replace the opto-mechanical scanning devices is the solid-state LiDAR. This LiDAR is based on a principle similar to phased arrays in radars. Phased arrays employ a number of transmitters which can change the direction of the laser beam, adjusting the relative phase of the signal from one transmitter to another. In addition to the optical phased array, there is also a micro-electromechanical system, which uses micro-mirrors for directional control and focus.

#### 2.2.3. Lidars with Diffractive Optical Elements

In these LiDARs, as in previous types, spatial scanning is carried out by laser light without moving parts. Panoramic scanning is achieved using phase-adjustable optical diffraction gratings (beam splitters) which divide the laser beam into a number of beams with a given spatial distribution. One can control direction, intensity, frequency, and phase features of the light radiation. This technology is widely used in the aerospace industry for remote scanning of the Earth, the Moon, and other planets.

Currently, self-driving solutions mainly use LiDARs with opto-mechanical scanning due to a greater coverage of the scanning area. Therefore, the physical principles for these types of LiDARs that can affect the detection of obstacles by the AVs will be discussed below.

### 2.3. Calculating the Distance from the LiDAR to the Object

The two most commonly used methods for object range determination are time-of-flight and phase comparison, due to the simplicity of algorithm implementation and further calculation of methodological errors introduced when using these methods.

#### 2.3.1. Time-of-Flight Methodology

The sending of probing pulses as a single pulse is propagated by a coherent LiDAR in the atmosphere is given by the following expression:

(1) $$T_{p}=\frac{1}{f_{p}},$$

where *T_p_* is the pulse repetition period and *f_p_* the pulse repetition frequency.

In between *T_p_* time period, the backscatter signal is received at distances of *R*, not exceeding the value:

(2) $$R_{max}=\frac{c\left(T_{p}-\tau_{p}\right)}{2n},$$

where

*c*—speed of light;
*τ_p_*—probe pulse duration;
*n*—is the refractive index of the transmission medium (taken as 1 for air).

During the *T_p_* time at the output of the analog-to-digital converter with bandwidth *BW* we get an array of LiDAR signal samples:

*X* (*mt_s_*; *n*), (3)

where *m* = 0, 1, 2, 3, …, *M* − 1—reference number;

*n* = 1, 2, 3, …—the number of the probing pulse packet sent into the atmosphere;

*t_s_*—reading time interval.

During the time *t_s_*, the pulse will move the distance determined by the following expression:

(4) δR=cts2.

#### 2.3.2. Phase Comparison Methodology

This technique is based on the previous one, the only difference is that the phase difference, rather than the time between the probing pulse and the incoming pulse, is considered.

In the same amount of time *t_s_,* the phase of the probing laser pulse travelling from the source to the object and back will change by the amount of *φ*:

*φ* = 2π*f_p_t_s_* (5)

where *t_s_*—reading time interval;

*f_p_*—pulse repetition frequency;

*φ*—phase difference between the probing pulse and the incoming pulse.

In this way, it is possible to determine the distance to an object:

(6) δR=cφ2πfp.

Considering the above description, one can assume which sources of interference may be contributing to increasing the noise in the detector and the creation of false alarms:

- temperature increase/decrease;
- high humidity;
- dust particles in the atmosphere;
- scattered radiation from other laser sources located within reach;
- direct radiation from other laser radiation sources located within reach.

## 3. Intentional Impacts on the LiDARs

### 3.1. Classification of Attacks on AV LiDARs

Analysis of scientific sources has shown that there are different approaches to classifying attacks on automotive LiDARs.

Thus, Shin et al. [8] give a general classification of attacks on sensors based on the exploited channel dividing them into regular, side channel, and transmission attacks. Regular channel attacks exploit the same physical parameters that are used by the target sensor; transmission channel attacks utilize the entities integrating the system and the sensor as its part; and side channel attacks can use any physical quantities, except for the ones operated by the target sensor. A similar approach was presented by Changalvala and Malik in paper [9], dealing exclusively with attacks on LiDARs of the autonomous vehicles, with exposure levels stratification. Only regular-channel (sensor level) and transmission-channel (interface level) attacks are distinguished, but their further implementations (among these two attack classes) are present: saturation and spoofing attacks refer to regular-channel, while point cloud manipulation to transmission-channel attacks. Spoofing attacks are divided into relay and replay attacks. Linking cloud point manipulation to transmission-channel attacks is not accidental. Deliberate malicious impact on the LiDAR and the advanced driver-assistance system (ADAS) is assumed to come from inside the AV. The authors indicate two main implementations of transmission-channel attacks, namely fake object insertion and target object deletion, the essence of which is obvious from their names and related to the manipulation of a target object’s points in the LiDAR cloud. Channel-based splitting of the attacks on the LiDAR is also mentioned by Shin et al. in [10]: sensor saturating and sensor spoofing attacks are certainly included in the regular-channel attacks group.

Petit et al. in [11] divide the attacks on LiDARs into front/rear/side attacks (the attacker influences the target LiDAR with equipment mounted on the other AV), roadside attack (the attacker impacts the target LiDAR with stationary tools on the road’s side), and Evil Mechanic attack (the target LiDAR is attacked from inside the AV, commonly via its interface). It can be assumed that the taxonomy of the attacks on LiDAR was carried out on the basis of the location and conditions used for the attack.

Jagielski et al. [12], considering attacks on the cooperative adaptive cruise control (CACC) system, suggested that the attacker’s goal is deceiving the AV’s radar, LiDAR or both of the sensors simultaneously. Attacks were considered on the basis of motion values, which were negatively affected. In particular, POS and VEL-POS attacks concerning the LiDARs were specified. In the first attack, the attacker changes the output of the LiDAR on the position of the AV, the second attack implies also the alteration of the radar’s output on the speed of movement.

Another unique approach to classification is related to the type of manipulation performed on the cloud of points, regardless of how such an operation is performed, as presented by Bahirat and Prabhakara in [13]. The additive approaches attack (adding new points of a fake object to the cloud), subtractive approaches attack (removing points from the cloud to hide the object), and deforming approaches attack (changing the location of the points to distort the shape and/or the size of the object) are distinguished. Two possible implementations of the additive approach attacks, namely copy-transform-paste and copy-re-sample-transform-paste attacks, are described.

A more common approach to classifying attacks on LiDARs involves dividing them on the basis of the method of implementation (or by the type of attack). Examples include:

- Cao et al. [14] distinguish LiDAR spoofing attacks (injecting fake points in the LiDAR’s cloud using lasers; emergency brake attack, and AV freezing attack as particular implementations) and adversarial machine learning attacks (crafting and putting object on the scene to deceive the LiDAR’s detection system);
- In addition to classifying attacks by type of points manipulation, Bahirat and Prabhakara [13] specify relaying and spoofing attacks;
- Stottelaar [15] describes jamming, relaying, spoofing, and denial-of-service (DoS) attacks;
- Shin et al. [10] classify sensor spoofing, spoofing by relaying, and sensor saturating attacks. Sensor saturating attack is also called blinding attack and has specific scenarios that can also be considered as its implementations: weak light source, direct strong light source, and oblique strong light source saturating attacks;
- Hau et al. [16] refer to LiDAR spoofing, object hiding, as well as object removal attacks;
- Petit et al. [11], considering attacks on LiDARs separately, describe relaying and spoofing attacks;
- Liu and Park [17] and Parkinson et al. [18] denote spoofing, and saturation (jamming) attacks;
- El-Rewini et al. [19] distinguish replay, relay, blinding, spoofing, jamming, and DoS spoofing attacks;
- Among the implementations of spoofing attacks, Rivera et al. [20] singled out several in terms of the impact on the LiDAR’s state: percentage spoofing attack, value spoofing attack, rotation spoofing attack, zero replacement spoofing attack, NaN replacement spoofing attack, repeated data spoofing attack, window repeated data spoofing attack, sector value spoofing attack, real world spoofing attack, and Frog Boiling spoofing attack. They can also be considered as specific implementations of a spoofing attack (highlighting the influence of the LiDAR’s state as a separate basis for the classification is not advisable due to considering different variants of only one type of attack).

The Adv-LiDAR approach, described by Sun et al. [21], combines features and mechanisms of both spoofing and adversarial machine learning, which does not allow to relate it to a particular attack type. This attack method shall be described separately.

**Figure 1:**
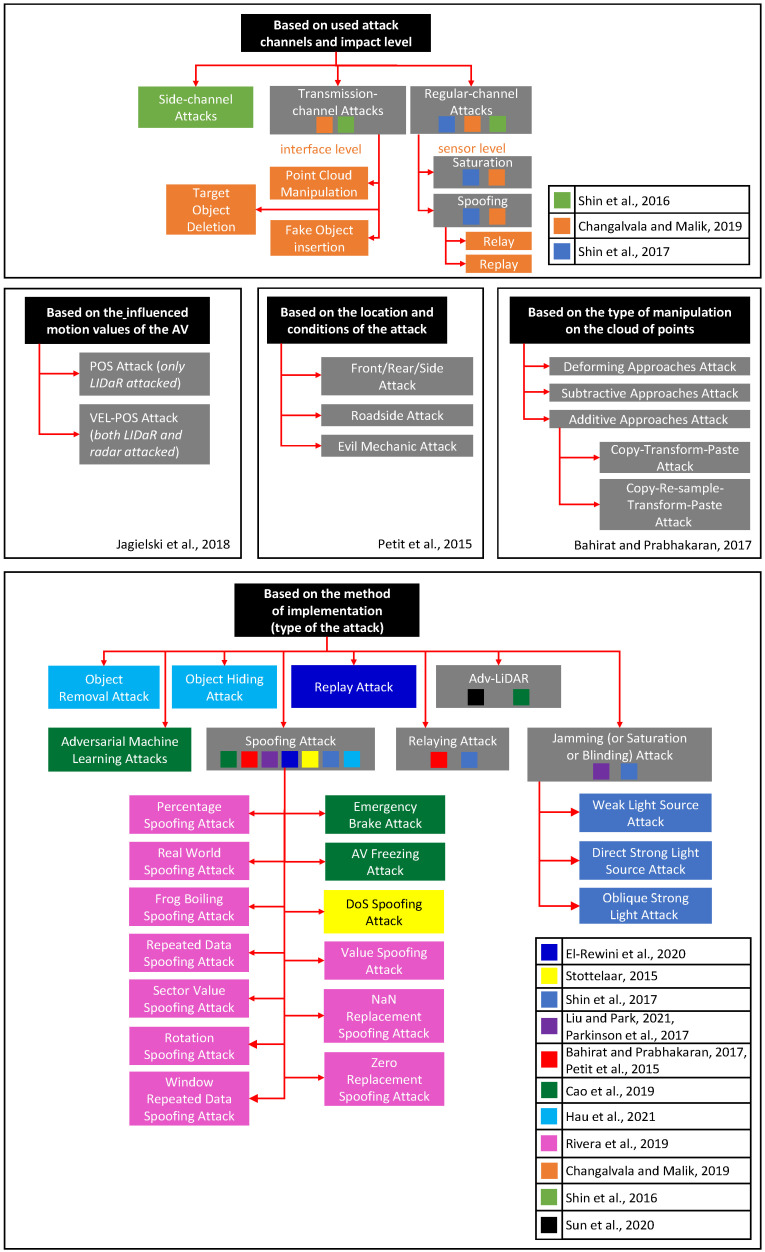
Approaches to classifying the attacks on automotive LiDARs.

Figure 1 presents the approaches used in [8,9,10,11,12,13,14,15,16,17,18,19,20,21] to classify attacks on LiDARs, as well as the corresponding bases indicated in the above sources. These attacks are described in more detail in the following section.

### 3.2. An Overview of Current Attack Vectors on the Automotive LiDARs

#### 3.2.1. Adversarial Attack

The essence of the attack lies in the concealed perturbations in the points cloud (the input 3D data of the object) carried out by the attacker, which lead to incorrect detection and/or recognition of the objects, or even the inability to identify and/or classify them. This type of attack is also called adversarial machine learning, as it is aimed precisely at deceiving the algorithms and methods of machine learning used that are sensitive to distortions or artificial perturbations [22].

In particular, paper [23] introduced a number of algorithms to form adversarial points in the cloud, which were then used for processing in PointNet deep neural network (DNN). Two subspecies of these attacks are given: shifting the positions of the existing 3-D points (adversarial point perturbation), and generating new points or point clusters (adversarial point generation). Adversarial point generation attacks included adding adversarial points, adversarial point clusters, or even adversarial objects. A more than 99% attack success rate has been declared.

A similar study was made by the authors in [24]. The work demonstrated that placing an adversarial object on top of the AV’s roof allows to fool the LiDAR detection system with a success rate of 80%, resulting in the failure to recognize the object as an AV (car). The difference from [23] is the greater universality of the adversary, as well as interaction with the real world on the scene. The practical use of objects such as those used in the study and their placement on the roof of the vehicle can prevent the LiDAR from detecting and recognizing it, and cause serious traffic accidents. Based on [16], this attack can also be called as adversarial machine learning object hiding attack.

Another implementation of adversarial attacks is the creation of specific adversarial objects, similar to ordinary physical objects, and their placement on the scene. The shape of the objects is chosen in such a way that their renderer in the form of an input cloud of points prevents the LiDAR from detecting and identifying them. An example and description of such an attack is presented in [25]. In particular, two methods to fool the LiDAR’s detection systems have been proposed: the evolution-based black-box attack method, as well as the white-box LiDAR-Adv approach based on solving the problem of optimization to create real adversarial objects. It should be noted that LiDAR-Adv has shown a higher success rate than the black-box approach (51%/71% versus 62%/36%) while using adversarial objects of various sizes with the Baidu Apollo driving platform. In addition, real tests with printed three-dimensional adversarial objects have been conducted. They confirmed the hypothesis that such objects allow to evade LiDAR’s detection (the experiments used Velodyne HDL-64E).

Adversarial attack on multi-modal 3D detection models (or multi-modal camera-LiDAR adversarial attack). Multi-modal 3D detection models use images and point clouds as their input, both separately (cascaded model) and simultaneously (fusion models). This is due to the fact that cameras and LiDARs on board of the AVs are mostly used to detect three-dimensional objects. During the attack, the authors [26] carried out perturbation of the shape and texture of the 3D object. The introduction of an adversarial object on a vehicle leads to the malfunctions of the DNN-based detection system. This approach can also be used in adversarial training [27]. The use of this type of attack gives a 50% chance to cheat both the LiDAR’s detection system and the cameras, so that the host vehicle will not be recognized (KITTI benchmark has been used by the authors to estimate the effectiveness of such attacks).

The Adv-LiDAR approach, presented in [21], also implies the use of adversarial machine learning techniques, but focuses mainly on the injection of fake point into the LiDAR’s cloud. Therefore, Adv-LiDAR will be the described separately.

#### 3.2.2. Spoofing Attacks

The essence of the attack is deceiving the target sensor through sending a fake signal (inject fake echoes) to it. This leads to misperception and incorrect mapping of the environment and/or detection of non-existing objects by the LiDAR (by forming fake points/clouds of points the attacker can both create virtual obstacles and virtually change the position of the existing ones). The attack is realized by sending a fake laser pulse during the listening window of the LiDAR to swap the location of the point(s); it will be assumed to be further away) [28]. In the simplest case, photodiodes synchronized with the target LIDAR are used: within a certain period of time the laser injects the fake points formed during the LiDAR’s detection cycles [17]. The feature of this type of attack is that the target LiDAR cannot physically (without the use of additional hardware or software algorithms) distinguish the attacker’s signal from the real reflected one, as they have the same physical channels [21].

Paper [16] singles out a separate type of attack, namely the object removal attack, whereby an attacker shifts the point cloud by introducing new points in the region of interest (i.e., within the bounding box area of the target 3D object, at some distance from the real cloud of points). The purpose of the attacker is precisely the incorrect detection of the target object.

The authors in [12] consider the attacks on CACC, when AVs perform tasks either individually or within a group (fleet/swarm). In particular, the paper describes two spoofing-based attack that imply LiDAR attack, radar attack and an attack on both sensors simultaneously. Two attack scenarios for LiDARs are as follows:

- POS attack: Implementing LiDAR spoofing to deceive the AV’s position in space.
- VEL-POS attack: A POS attack simultaneously spoofing AV’s speed values (radar attack).

Lidar signal replay attack and signal relay attack are both considered as variations of spoofing attacks.

1. Signal replay attack. The attacker constantly receives and records LiDAR signals and then sends them back, resulting in forming a cloud of points for a nonexistent object [15,19,29]. Another example of such an attack is the study [30] where the authors used Raspberry Pi and a low-power laser to compromise the LiDAR, as well as to create a fake obstacle for the AV, which led to its emergency braking.
2. Signal relay attack (or “spoofing by relaying” as stated in [10]). The LiDAR signal is received by the attacker (photodiode is usually used for synchronization purpose), recorded and transmitted to a transceiver located elsewhere from where the signal is transmitted back to LiDAR (with a certain and configurable delay) using an “attack” laser triggered by a specially crafted pulse waveform. This creates fake echoes. By changing the delay value, the attacker can “select” fake points to be inserted into the point cloud. Such an attack can lead to distortion in mapping of the objects (real objects will be further or closer to their respective cloud of points formed by the LiDAR). For example, in the work [11], the cheap Osram SFH-213 photodetector and Osram SPL-PL90 laser were used to conduct such an attack (target LiDAR—ibeo LUX 3). As a result of injection of counterfeit points into the point cloud, the LiDAR detected a non-existing wall.
3. In [10], a signal relay spoofing attack, experimentally verified using the Velodyne’s VLP-16 LiDAR, has been described and carried out. The photodiode and pulsed laser diode used are similar to [11]. The attack to spoof the LiDAR created an obstacle that was closer to the AV than the location of the attacker (the device used to a spoof the LiDAR).
4. Adv-LiDAR. Due to the wide range of considered issues, and the use of a new type of LiDAR vulnerability (ignoring occlusion information in the point clouds by the LiDAR’s perception models), results obtained by Cao et al. [14] should be regarded as a separate spoofing attack implementation. The authors managed to create about 100 fake points at once at a distance of more than 10 m, and algorithm presented in the work allowed to increase the success rates on average 2.65 times (in Baidu Apollo LIDAR perception module, overall attack success rate of 75%). It is assumed that the attacker can act both independently (as a pedestrian influencing the AV) and while inside the other AV moving in the adjacent traffic lane. As a result of the attack, the target AV had to stop abruptly (from 43 km/h to 0 km/h in 1 s), which in real-world conditions could lead to serious consequences. The proposed Adv-LiDAR approach in addition to spoofing attack also used adversarial machine learning methods to deceive the very algorithm of LiDAR’s perception (experiments based on the Baidu Apollo 3.0 model and their Sim-control AV simulator, known photodiode and a laser diode [10,11] were used to physically attack the LiDAR, the “victim” was Velodyne’s VLP-16 PUCK LiDAR). Thus, Adv-LiDAR includes both spoofing attack and adversarial machine learning attack techniques, and thus belongs to both types of the attacks on automotive LiDARs.

The paper also considered two spoofing attack scenarios that can be distinguished as separate attack subspecies:

- Emergency brake attack. Adversarial 3D point cloud creates spoofed obstacle that is in “located” in front of the AV, in close proximity to it. This forces the AV’s self-driving system to use emergency brake system to avoid a fake obstacle;
- AV freezing attack. A front-side obstacle is spoofed by creating an adversarial 3D point cloud in front of the AV (and in close proximity to it). In the case of being in a stationary position at the time of the attack, the AV will not be unable to start moving.

5. DoS spoofing attack. In papers [15,19], the DoS attack stands out as one of the most appropriate implementations for this type of attack. It is assumed that the attacker’s objective is to spoof the LiDAR via the injection as many fake points as possible. This will result in ample false 3D objects to exceed the maximum number of trackable objects of the target LiDAR.
6. As mentioned earlier, the authors of [20] divide the spoofing attack based on the effect on the current state of the LiDAR, since the attack vectors are irrelevant. In particular, the article singles out the following attacks (given with a short description corresponding to the content of the work [20]):
  - percentage spoofing attack: increasing input values by a specified percentage;
  - value spoofing attack: shifting points in the cloud via a given value;
  - rotation spoofing attack: changing only the indices of the measured values array;
  - zero replacement spoofing attack and NaN replacement spoofing attack;
  - repeated data spoofing attack: looping the replay of the input;
  - window repeated data spoofing attack: looping the repeat of some value (or a number of values) as an input in a form of a sliding window;
  - sector value spoofing attack: relay of a fake LiDAR signal from a certain direction;
  - real world spoofing attack: only part of the LiDAR point cloud is affected, which corresponds to actual external attacks using additional equipment (e.g., laser);
  - Frog Boiling spoofing attack: scrupulous change of the LiDAR’s input (one or several points in the cloud) below the detection filter (threshold value) to avoid attack detection (attack named in accordance with [31]).

#### 3.2.3. Saturation Attack

Forcing the LiDAR into a saturation region occurs when a sufficiently powerful laser beam with the same wavelength is directed to the LiDAR. If the receiver of the LiDAR is saturated, it is unable to acquire the reflected echoes. This results in “ignoring” the objects around the AV, located in a certain direction; these objects will not be reflected in the LiDAR’s point cloud. Due to this feature, this class of attacks is also called blinding attacks (e.g., in [10]) or jamming attacks [18]. The objective of the attacker can be hiding objects, LiDAR’s DoS, or both [17,20]. It should be noted that when using direct strong light source, the direction in which the LiDAR gets “blind” will correspond to the direction from the LiDAR to that light source. In addition, a cloud of fake points may also appear as a result of noise effects during such attacks (in case of a direct ray beam, the location of fake points will correlate with the direction to the attacker’s light source).

The study [10] is an example. Two 905 nm laser modules were used for saturation attack experiments: 30 mW (weak light source), and a power-adjustable 800 mW one (strong light source), target LiDAR was Velodyne’s VLP-16. Three scenarios have been considered for weak and strong light source (both direct beam and oblique ray directions). The results of the experiments were consistent with the general description of the attacks: multiple fake points occurrences in all scenarios and sectoral dazzle of the LiDAR under strong light exposure.

#### 3.2.4. Other Attacks on the LiDAR

1. Cloud points manipulations using raw data. The authors [13] distinguish three groups of forgery attacks on the LiDAR, based on the method of changes: additive approaches attacks, subtractive approaches attack, and deforming approaches attack. The additive approaches attacks imply adding new points to create an object on the scene, subtractive approaches attack—removing the points that make up an object to delete it from the scene, while in the deforming approaches attack the surface and/or the shape of the object are deformed by altering the position of a number of points. The points’ position shift may lead to incorrect detection of the object or even its non-detection by the LiDAR. Two implementations of additive approaches attacks are given: a copy-transform-paste attack and a copy-re-sample-transform-paste attack. The latter one allows matching the forged object to the LiDAR’s resolution to be placed in the dot cloud (on the stage) properly. The proposed attacks are similar to both adversarial and spoofing ones. Despite this, these attacks cannot be defined as spoofing attacks, as the paper explicitly states that no additional hardware is required for these attacks, i.e., the creation/removal/changing of the points in the cloud cannot be performed via sending fake signal to the LiDAR. In addition, there is also no indication that the described attacks are intended to be used to mislead recognition and machine learning systems, which also prevents them from being classified as adversarial machine learning attacks. Thus, the described attacks are introduced into a separate class. As stated in Section 2.1, this type of attack belongs to transmission-channel ones.
2. Using objects with surfaces of extremely high reflectivity or absorptive capacity. Petit et.al. suggested to use special surfaces with increased reflection or absorption values for infrared light, which can lead to the formation of a fake cloud of points, hinder the detection of objects with distorted shapes and dimensions or total/partial malfunction of LiDAR’s detection system [32]. Still on this topic, over the last few years, materials and paints with extremely high reflectivity values have been created, e.g., (but not limited to) Vantablack and Vantablack 2.0 [33], Black 3.0 [34], Musou Black, “dark chamaleon dimers”, etc. For example, the latter, being a nanostructured material, can absorb up to 98–99% of light rays in 400–1400 nm wavelength range, with no relevance to the angle and polarization type of falling light [35], and the BMW managed to introduce their X6 car coated with Vantablack [36]. Considering that LiDARs mostly function at 905, 940, and 1550 nm wavelengths [37], the studies of LiDAR behavior in the presence of objects (or cars) with the above materials and paints (or coatings with high reflectivity values) should be relevant. Whether such objects pose a threat to LiDARs and/or AV’s ADAS remains an open question (at least in this paper).
3. Side-channel attacks. Such attacks are based on receiving (extracting/intercepting) the useful signal due to the physical implementation of target systems, the generated physical fields, or the physics of the processes occurring therein. Possible LiDAR attacks can be based on timing, power consumption, and electromagnetic emissions. For example, it can be assumed that since the LiDAR converts the value of laser pulse intensity into electrical signals [17] and contains electronic components, it should be susceptible to leaks of useful signals through various types of parasitic emissions of its electronic equipment (TEMPEST), while small fluctuations of the voltage consumed may be correlated with the features of microcontrollers and electronic control units (ECUs). In the first case, the attacker can intercept the compromising electromagnetic radiation and extract the useful component of the signal. In the second case, they can figure out the logic and the time parameters of the computational operations performed. We failed to find any open-source studies concerning the implementation of side-channel attacks on the automotive LiDARs. Despite this, the study of LiDAR side-channel attacks seems to be relevant.
4. Lidar’s ECU malware attack. Infection of the ECU via the malware can cause both DoS of the LiDAR (or the whole AV at once) and malicious disturbances in its functioning [22]. Such an attack can be carried out if the intruder has physical access to the AV (e.g., Evil Mechanic attack [11]) or through the adversary impact on the ECU (for example, when upgrading firmware; over-the-air attack) [38]. Such attacks can seriously compromise the safety of the autonomous vehicles, transported passengers or cargo, and other road users.
5. Evil sensor calibrator and data fusion manipulator attacks. Navigational data fusion algorithms are designed to provide redundant information on AV motion parameters, its position, and the characteristics of other traffic participants, as well as the infrastructure of a “smart city” (in both local and global coordinate systems). In turn, sensor calibration and pairing procedures are designed to compare data from different sensors and systems for further processing, including to ensure mutual correction and correct operation of the AV in cases when one or more sensors have failed. An attacker may interfere with the calibration process of a pair of sensors (evil sensor calibrator attack), one of which is LiDAR (e.g., LiDAR-GNSS, LiDAR-IMU, LiDAR-camera, etc.), or the algorithms of data fusion in the navigation subsystem (call it fusion data manipulation attack). As a result, the algorithms and methods used in the LiDAR or its ECU to counteract spoofing or other adverse effects (if any) will not be effective, as correcting the LiDAR readings from other sensors and systems will not be possible. The resilience of the AV’s navigation system will be impaired, and ECU will receive false information about traffic parameters and the location of the AV on the road, which could lead to accidents [38].

## 4. Unintentional Adverse Impact on the LiDARs

### 4.1. Classification of Unintentional Impacts on LiDARs and Their Sources

External sources of unintentional adverse impact on lidars can be classified into three main groups: other LiDARs, other in-band light sources, and environmental conditions (harsh weather and solar radiation) [39]. The authors use different taxonomies of the types of LiDARs’ mutual interference. In particular, Hwang et al. [40] describe transient and in-band types of mutual LiDAR interference.

On the other hand, Popko provides direct (direct signal from one LiDAR to another) and scattered (the arrival of a reflected signal from another LiDAR) interference. The author further divides direct interference by diffuse-direct and coupled-direct [39] types.

In addition, Godbaz et al. [41] identified multipath interference as its separate type, when a signal from other LiDAR arrives at a receiver of the target LiDAR via two or more routes, simulating multipath interference for the amplitude-modulated continuous wave LiDARs.

Full understanding of the nature of LiDARs’ mutual interference requires an analysis of the available researches with interference theories.

### 4.2. LiDAR Mutual Interference Theories

Using several LiDARs will cause interference, particularly in the case of LiDARs with common angles of scanning. The sphere of research on the influence of multi-LiDAR interference is especially relevant for the automotive LiDARs, especially in the context of their use on the UVs.

Kim et al. [42] use a geometric approach to the LiDAR mutual interference problem and indicate multipath propagation interference of multiple LiDARs among the possible scenarios. In an experiment [43], the authors have demonstrated the presence of interference from two simultaneously operating 905-nm pulsed ToF LiDARs in face-to-face and two side-by-side arrangement schemes, as well when placing two LiDARs in back-to-back position, in paper [44]. In the work [43], the authors have also carried out an in-depth analysis of the experimental data, indicating the successive interfered angles, as well as the general rate of mutual successional interference in different scenarios of LiDAR installation. Although there is clear evidence of a problem of mutual interference, the authors do not provide a general mathematical theory. Further development and generalization of the authors’ approach has been conducted in papers [45,46]. Kim et al. also proposed the use of the pulsed scanning LIDAR with direct sequence optical code division multiple access modulated signal as a means of dealing with mutual interference [47].

The geometric approach to the description of LiDAR interference was also presented in the work of Popko [39]. A mathematical model of LiDAR interference, its sources and classification, as well as the results of a series of simulations and real experiments (with adequate comparison of the obtained data) in different LiDAR placement schemes were presented. Geometric approach for optical ray tracing and the estimates of LiDAR mutual interference continued in work by Popko et al. for the in-plane scheme with two LiDARs [48]. Despite the solidity of the theory formed and the results presented, the described model is not comprehensive. In particular, there is no mathematical description of the influence of arbitrary configurations (for example, rotating ToF LiDARs with non-parallel rotation axes) on the mutual interference, and there is no optical radiation theory application for arbitrary wavelengths with both direct and scattered interference. Moreover, the proposed theory of LiDARs interference is based on the efficient geometric approach and Monte Carlo techniques when considering LiDARs’ beam intersection. However, it is unlikely that radiometry methods will be completely abandoned when considering the interference of moving UV-based automotive LiDARs.

Hwang et al. considered the problem of interference in the frequency-modulated continuous-wave LiDARs. As in other cases, their results show that the interference reduces the SNR and brings extraneous objects in the point cloud [40].

Mutual interference theory with considering several LiDARs was introduced by Zhang et al. [49] in terms of the LiDARs’ equations and their ranges, as well as the number of photons sent/received, and the detection probability (but only for Geiger-mode avalanche photodiode-based LiDARs). Thus, it may be difficult to adapt this theory for other (more common) LiDAR systems such as single-photon avalanche diodes (SPAD-based) ones.

In addition, the approach to the LiDAR interference description, as well as the proposal to address the problem by using the developed interference filtering algorithm were given by Diehm et al. in paper [50].

### 4.3. The Influence of Harsh Weather and the Environment on the LiDARs

The approach to the detection of adverse weather conditions for the operation of the LiDARs has been considered by Heinzler et al. [3]. The experimental use of the feature vector approach with Velodyne VLP16 and Valeo Scala LiDARs allowed to create an efficient harsh weather detection algorithm. The work implied the use of comprehensive datasets acquired from the experiments in a real climate chamber. Despite this, only the influence of fog on automotive LiDARs has been considered.

Lin and Wu [4] proposed the use of the combination of the nearest neighbor segmentation algorithm and the Kalman filter for noise filtering purposes. Significant improvement in LiDAR detection (Velodyne LiDAR PUCK VLP-16) has been achieved (given as the root mean square error): 11–16% in rain and smoke, 15–23% in smoky weather, and 33–35% for rainy conditions. However, the paper does not assess the applicability of the approach to real conditions (several UVs on the road in harsh weather conditions). In addition, the impact of additional resource-intense computing on the framerate of the point cloud has not been investigated. Charron, Phillips, and Waslander proposed the use of the dynamic radius outlier removal filter to remove noise caused by snow [5]. The used approach is much more efficient than the analyzed 2D and 3D filters. Snow-induced noise filtering in the LiDAR occurs during the processing of the existing point cloud. However, additional full-scale testing is required. Furthermore, the applicability of the proposed approach to other adverse weather conditions (rain, fog, smoke, etc.) remains unclear. Wu et al. [51] use the point density filtering method with enhanced DBSCAN algorithm to improve the performance of the LiDAR in windy and snowy weather, but for the case of foggy weather further research on the applicability of this approach is also required.

Kutila et al. in paper [52] showed, that LIDAR performance in simultaneous fog and snow conditions in winter can drop up to 25%, and the range of the detection of objects can decrease up to three times. Despite several tests with the Ibeo LUX LiDAR, the overall study focuses on detecting the presence of adverse weather conditions (fog, snow, or rain) and assessing their impact on the LIDAR, and the influence of fog and snow on the overall efficiency of the detection of obstacles of the UV when moving was not considered. Jokela et al. analyzed the behavior of the LiDARs in arctic weather conditions [53]. The study included an assessment of the influence of high air humidity, wet road surface, low temperatures and snowstorms on Cepton HR80T, Ibeo Lux, Velodyne Puck VLP-16, and Robosense RS-LiDAR-32 LiDARs. In particular, Cepton HR80T and Robosense RS-LiDAR-32 LiDARs start to fail at −5 °C, and at below −10 °C data from these LiDARs become useless. In addition, the wet surface and blizzard cause echoes and perturbations in the point cloud. Park et al. proposed an intensity criteria-based denoising technique using a low-intensity outlier removal filter to mitigate the impact of snow particles on the LiDAR’s cloud of points, which allows to increase the speed of processing data [54].

Ronen, Agassi and Yaron [55] used the polarization method to reduce atmospheric light backscattering for the LiDAR, proposing a model of LIDAR functioning deterioration in the foggy and cloudy conditions (based on Mueller matrix and modified Stokes vectors).

Rivero et al. [56] proposed a model for training neural networks for detecting the influence of spray water on the LiDAR’s point cloud based on a synthesized dataset.

Trierweiler et al. [57] investigated the degradation of LiDAR efficiency due to the pollution of its cover glass. In particular, it has been determined that the maximum range of detection of objects can be reduced up to 75%. This sphere of research is expected to be promising because the automotive LiDARs will have to operate properly in polluted conditions.

## 5. Improving LiDAR’s Security and Resilience by Mitigating Negative External Impacts

### 5.1. Increasing the Resilience to Mutual LiDAR Interference

Below are the approaches, methods, algorithms, technologies, and solutions proposed by various authors to increase the robustness and the resilience of LiDAR to the impact of mutual interference.

In paper [58], Seo et al. proposed the 36-channel SPAD-based scanning-type LiDAR using matching peaks pairs-based interference filter to acquire valid ToF values. Despite providing good practical results, the presented MEMS-based scanning LiDAR may require additional tests, including for interference resilience when used on the UVs. In addition, there is no data on whether the adaptation of the proposed technology is planned for other main LiDAR frequencies (940 and 1550 nm).

A similar approach to considering the LIDAR interference (both unintentional and intentional) has been proposed by Carrara and Fiergolski [59] in the SPAD-based LiDAR structure by introducing time-correlated discrete random delays in the formation of laser pulse of the target LiDAR. Despite the obvious efficiency and simplicity of the proposed approach, the problem of considering the case when all other “adverse” LiDARs work with the same random time-shift scheme remains unsolved. In addition, as the number of UVs with LiDARs operating in close proximity to each other increases, so does the probability of synchronization of the send/receive events, even when a fixed time is used between the pulses, and the delays of the target LiDAR are true-random.

Hwang and Lee in a series of papers [60,61] proposed to use their version of true-random LiDAR based on Gaussian signal generator that showed good SNR values. In addition, it has been experimentally proven that there is no correlation between the interference power and the value of false response probability when LiDARs of different types (continuous-wave, pulse-modulated, and frequency-modulated) work together with the target one.

Lei et al. [62] proposed the use of arrays of TOF pixels of the octagon and central symmetric shape for the solid-state LiDAR of long range in order to enhance the resilience to the interference from other sources of light. Ximenes et al. [63] proposed a unique SPAD-based direct TOF image sensor for LiDAR capable of reducing interference.

Other analyzed solutions for suppression of mutual interference include:

- frequency-hopping modulated LiDAR for autonomous vehicles [64];
- FPGA-based dual-pulse LiDAR with digital chaotic pulse position modulation [65];
- CMOS LiDAR sensor with a SPAD-based random number generator and high background noise immunity to detect time-correlated TOF [66];
- chaotic laser imaging lidar based on the orthogonality of the Boolean chaotic waveform;
- SPAD-based LiDAR with photon-driven stochastic pulse position modulation [67];
- software-based optical sampling scheme for ToF LiDAR with pseudo-random binary sequences [68];
- interference-robust CDMA LiDAR [69];
- interference-resistant 3D Pulsed Chaos lidar [70].

### 5.2. Methods of Protection against LiDAR Attacks

The analyzed scientific sources suggest a number of methods and techniques for both detecting and countering attacks on AV LiDARs.

In particular, the main way to protect against adversarial attacks is to use robust models in both DNNs and LiDAR object detection systems (Abdelfattah [27]).

To counter spoofing and jamming attacks, researchers suggest different approaches that can be roughly divided into sensor level-based and data level-based ones. Sensor level-based approaches use hardware capabilities, for example:

- LiDAR signal modulation using a side channel analog signal to protect against false echoes (and false points in the cloud respectively; Matsumura et al. [71]);
- the use of signals with different wavelengths (Petit et al. [11]);
- dynamic change of laser pulses time interval (Parkinson et al. [18] and Takefuji [72]).

Examples of data-level-based protection against LiDAR spoofing are as follows:

- the use of density consistency check approach to detect false points in the cloud (Bahirat and Prabhakaran [13]);
- the use of 3D temporal consistency check to verify detected objects (You et al. [73]);
- watermark insertion into the data of the LiDAR to detect and localize fake object insertion and deletion (Changalvala and Malik [9]);
- utilizing vehicle-to-vehicle links to increase situational awareness using measurements of other AVs (Stottelaar [15]);
- LIFE (LIDAR and Image data Fusion for detecting perception Errors)—LiDAR and camera data comparison method based on spatial and temporal features to detect data anomaly (Liu and Park [17]);
- occlusion-aware hierarchy anomaly detection (CARLO)—an occlusion-based approach to detect laser spoofing attacks (Sun et al. [21]).

The idea of using an extended Kalman filter with AV position data obtained from the global navigation system (GPS) and the inertial navigation system allows to mitigate LiDAR replay attacks in multi-sensor AV systems (Liu et al. [29]) and increase the number of objects being simultaneously monitored by the LIDAR [15], to counter DoS spoofing attacks.

### 5.3. LiDAR Resilience Improvement via Increasing the SNR

The review shows that methods used to counter attacks on the LiDARs are generally divided into physical (where modulation types and optical frequency are modified, including through the use of frequency hopping, etc.) and software (by using anomaly detection, data integrity checking, improving the circuit processing of point clouds using better filters, etc.) ones. However, dealing with unintended impacts on the LiDARs in the analyzed studies is based on the prevailing physical/hardware approach, e.g., proposing new designs of lidars, introducing enhanced noise filtering elements, the use of different types of signal modulation, etc. However, as already mentioned in Section 1, physical approaches to improving the LiDAR’s resilience are expensive and labour-intensive, requiring considerable research and production capacity.

Mitigating the influence of interference of various kinds on the functioning of the LiDAR is inextricably linked to the increase of the SNR for the target object. This means that by solving this problem we will achieve the goal of increasing the overall robustness of LiDARs, and the use of SNR improvement techniques are no less relevant approaches to increasing the general resilience of the LiDAR systems, as well as to dealing with adverse objects in the point cloud as a result of interference. SNR at the output of the photodetector is the main parameter describing the performance of the LiDAR and the capacity of its receiver subsystem. In turn, the probability of a signal being detected is directly related to the SNR, the chosen threshold value for the detection of the reflected signal in the LiDAR, and the decision on the presence or absence of the input signal.

The probabilistic approach to estimating the allowed SNR for the LiDAR and probability of detecting a reflected pulse developed by the authors (and detailed in the following Section) is based on the mathematical statistics and the determination of the SNR threshold using the Neyman–Pearson criterion, as it does not require a priori information about surrounding objects and the detection probability characteristics. Given the known error distribution, it is possible to derive the error probability from the SNR by setting different threshold values. In addition, according to the Neyman–Pearson criterion, it is also possible to choose the value of the threshold at which a minimum probability of “skipping” an object when scanning is provided (given that the false alarm does not exceed some given value).

## 6. Proposed Method

### 6.1. Approach to Estimating the Probability of Detecting a Reflected Pulse in Space

Several studies [71,72] consider the possibility of mutual interference between LiDARs located within each other’s reach hindering the detection of the obstacles. However, the probability of these events is not considered. In this paper, we propose a probabilistic approach to calculate “false events” or, simply put, induced fake points when scanning space, taking into account the Gaussian distribution of interference. The described approach is based on the used method of determining the error probabilities in radiolocation.

A raw dataset consisting of all received signals (with noise constituents) is taken as the Input. Then, a set of error probability dependencies for different SNR threshold values will be returned as the output. We also introduce the following and limitations:

1. This approach has been tested using ideal environmental parameters (normal LIDAR operating conditions) and did not take into account the background changes of the environment. Further studies are expected to take into account background noise caused by sunlight in clear weather conditions.
2. The quality of the point cloud is not taken into account. Instead, the probability value of false alarm is stabilized in relation to the maximum probability of correct response. Since the probability of a false event occurrence is not known at every moment of time, it is possible to apply the Neyman–Pearson criterion when choosing the solution.
3. The approach does not allow to quantify the elements of various types of errors from the sources of uncertainty.
4. The study did not identify clearly defined sources of uncertainty in different LiDAR subsystems, only the resulting error has been estimated.
5. Errors caused by the environmental noise have not been into account separately, but their overall contribution to the background error has been considered.

### 6.2. Description of the Proposed Approach

First, it is necessary to form arrays of the measuring system’s states. In this case, we consider LiDAR under external distortions and in the absence of them. The way to generate such arrays is as follows:

1. The detection or extraction of an incoming pulse should be presented as the sum of all return pulses:
  (7) $$DImp=\left(d_{r_{j}}\cap d_{disr_{j}}\right),$$
  where *DImp*—is the generated set of detectable pulses, $d_{r_{j}}\in \left\{d_{r_{1}},\;d_{r_{2}},\;\ldots ,\;d_{r_{n}}\right\}$—the generated set of return pulses from the considered LiDAR;$d_{disr_{j}}\in \left\{d_{disr_{1}},\;d_{disr_{2}},\;\ldots ,\;d_{disr_{n}}\right\}$—generated set of pulses from the LiDARs located within reach of each other, as well as multiple pulse re-reflections from objects in space.
2. Generate a set of values to assess the probability of “false alarms”:
  - Boundary conditions for the interference of re-reflected laser pulses from the considered LiDAR are as follows:
    (8) $$\left\{d_{r_{j}}\in \left\{D_{r}\right\}|\;D_{interf}\cup Q,\;q_{k}\in \left\{Q\right\}\right\},$$
  - Boundary conditions for detection (extraction) of the probing pulse from LiDARs located within each other’s range:
    (9) $$\left\{d_{r_{j}}\in \left\{D_{r}\right\}|\;D_{p}\cup Q,\;q_{k}^{\prime }\in \left\{Q^{\prime }\right\}\right\},$$
  where *D_r_* is the generated set of return pulses from the LiDAR for the corresponding set *D_interf_*; *D_interf_* is the set of interference pulse values; *D_p_* the set of multiple probing pulses from LiDARs located within range of each other; *Q* the set of factors contributing the interference of re-reflected impulses; *Q*′ the set of conditions for detecting probing pulses from LiDARs located within reach of each other.
3. Definition of the transition matrix for “false event” detection:
  - for the interference of re-reflected laser pulses from the considered LiDAR:
    (10) $$\left\{\exists !\in \left\{S_{{d_{r}}_{j}}\right\}|\sum_{j=1}^{n}P_{ij}=0.05,q_{k}\right\},$$
  - to detect (isolate) a probing pulse from LiDARs located within each other’s range:
    (11) $$\left\{\exists !\in \left\{S_{d_{disr_{j}}}\right\}|\sum_{j=1}^{n}P_{ij}=0.05,q_{k}^{\prime }\right\},$$
  where $S_{{d_{r}}_{j}}$ is the set of estimates of the pulse parameter from the considered LiDAR, $S_{d_{disr_{j}}}$ the set of estimates of pulse parameters from LiDARs located within each other’s range; and $P_{ij}$ the probability of detecting a “false event” for each laser pulse under certain contributing factors and events (if the probability of correct detection of the returning laser pulse is assumed to be 0.95, then the probability of “false events” is 0.05).

After forming the arrays of states of the measuring system (the LiDAR) under different external disturbing influences, one can proceed to the next step.

If a simple concept is applied, where there are two possible events, a return pulse and a “false event”, and where the probabilities of these events are known, then a Bayesian method can be applied. However, more often the a priori information in space location is not known, and this requires to use the Neyman–Pearson criterion. In this approach, we fix the value of probability of detecting a “false event” (this probability value should not exceed 0.05). Probability *P_d_* of correct detection of return pulse from object by a LiDAR under Gaussian (normal) noise is calculated as follows:

(12) $$P_{d}=\Phi \left(\varpi -\varpi_{n}\right),$$

where Φ(*Z*) is the integral pulse detection probability distribution:

(13) $$\Phi \left(Z\right)=\frac{1}{\sqrt{2\pi }}\int_{-\infty }^{z}exp\left(-\frac{t^{2}}{2}\right)dt,$$

**Figure 2:**
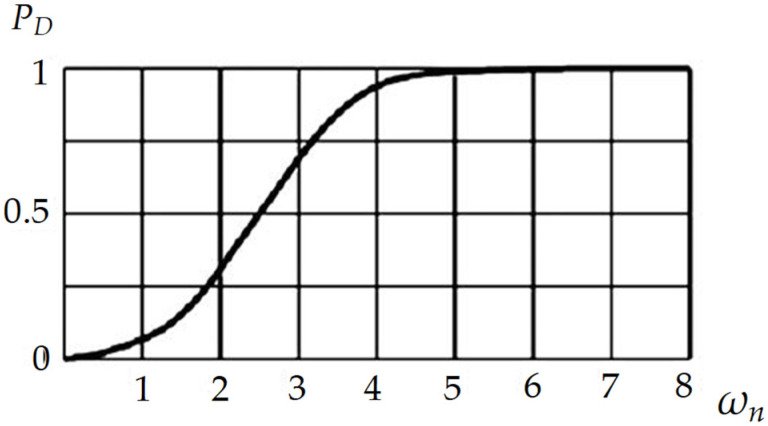
An example of return pulse detection probability as a function of the SNR.

If the relation between the probability of detecting a return pulse and the SNR (an example is shown in Figure 2) are known, it is possible to determine the maximum value of the noise.

### 6.3. Applying the Approach

Now introduce two hypotheses.

**Hypothesis** **1** **(H1).** *The received (detected) signal contains only noise.*

**Hypothesis** **2** **(H2).** *The received signal contains no noise.*

According to the description of the formulated binary problem, and known result of the receipt (only noise in hypothesis H1 or a useful signal in hypothesis H2), it is possible to describe the probabilities of occurrence of these events. Thus, the probability of the occurrence of a “false point” will be described as follows:

(14) PF=∬G0p(z¯H1)dz¯,

The probability of detecting a useful signal (pulse) is:

(15) PD=∬G1p(z¯H2)dz¯,

where $\bar{z}$ denotes all recorded observations;

p(z¯H1)—the density of distribution of the observations belonging to the hypothesis H1.

p(z¯H2)—the density of distribution of the observations belonging to the hypothesis H2;

These probabilities are not known a priori, so it is possible to apply the Neyman–Pearson criterion. To do this, we need to solve the problem of finding a conditional-free extremum using the Lagrangian function in the following form:

(16) Fλ=λPF0+∬G1(p(z¯H2)−λp(z¯H1))dz¯¯,

Using an approach similar to the Bayesian method, we determine the quality criteria. To do this, we can determine which points from the observation region $\bar{z}$ should be included in the region *G*_1_, so that $F_{\lambda }$ is maximal. If the integrand at some observation point is positive, then the observation point should be included in *G*_1_ If the integrand is negative, the point from $\bar{z}$ should be included in the region *G*_0_.

Write down the condition as formulated above:

(17) $$p\left(\bar{z}∕H1\right)>p\left(\bar{z}/H2\right)\to H2\;\Lambda =\frac{p\left(z∕H2\right)}{p\left(\bar{z}∕H1\right)}\;\{\begin{array}{c} <\lambda \to H2\\ \leq \lambda \to H1 \end{array}.$$

This is the optimal algorithm according to the Neyman–Pearson criterion. Using this algorithm, the maximum probability of correct detection with a fixed probability of false alarms can be obtained as:

(18) $$\int_{\lambda }^{\infty }p\left(\lambda ∕H2\right)=P_{F_{0}},$$

where $P_{F_{0}}$ is a fixed value for the probability of a “false alarm”.

The probability threshold for a false alarm will be determined as:

(19) $$P_{F}=P_{F_{0}}\;\int_{\lambda }^{\infty }\omega \left(\Lambda ∕H1\right)\alpha \Lambda =P_{F_{0}}.$$

Consider applying the algorithm described above to determine the probability of a false alarm from the LiDAR signal power. It is assumed that the LiDAR is mounted on a moving object (AV), so the individual signal powers are more likely to be distributed according to Rayleigh’s law.

Write down the Rayleigh distribution density:

(20) $$p\left({\bar{z}}_{i}|H2\right)=\frac{z_{i}}{\sigma^{2}\left(1+q\right)}e^{-\left(\frac{z_{i}^{e}}{2\sigma^{2}\left(1+q\right)}\right)},$$

where $q=\frac{\sigma_{signal}}{\sigma_{noise}}$—is the value of the signal to noise ratio;

*σ*—scale factor.

Using the likelihood ratio, write down the joint density as follows:

(21) ω(z¯H2)=∏i=1nziσ2n(1+q)e−12σ(1+q)∑i=1nzi.

Introduce a variable:

(22) $$T=\sum_{i=1}^{n}z_{i}^{2},$$

where *T* is the sum of observations.

For this variable, we describe the distribution density when there is no signal by the expression:

(23) $$\omega \left(-\bar{z}∕H1\right)=\frac{\prod_{i=1}^{n}z_{i}}{\sigma^{2n}}e^{-\frac{1}{2\sigma^{2}}T}\;\Lambda =\frac{1}{{\left(1+q\right)}^{n}}e^{\frac{1}{2\sigma^{2}}\left(\frac{q}{1+q}\right)T},$$

Since $\left(\frac{q}{1+q}\right)$ and (1+q) values are known, testing the hypothesis of maximum detection is:

(24) $$T\{\begin{array}{c} >T_{0}\to H2\;\left(the\;signal\;is\;present\right)\\ \leq T_{0}\to H1\;\left(no\;signal\right) \end{array}.$$

According to the condition written above, the sum of the observations must be compared with the SNR threshold:

(25) $$\sum_{i=1}^{n}z_{i}^{2}≷T_{0}.$$

We use the following assumption: the sum of a large number of small values is distributed according to a normal law. Using the known density of the “false points” distribution, we select the quantile and determine the threshold for the SNR.

The main stages of the proposed approach can be presented as an algorithm for finding the dependence of correct signal detection from “false alarms” that is shown in Algorithm 1.

### 6.4. Generating Datasets for the Research

In remote spatial scanning, as well as the development of optoelectronic systems of the LiDARs, the following equation can be used as a mathematical representation of the laser signal [74]:

(26) $$P_{r}\left(\lambda ,r\right)=\eta_{all}\eta_{g}\left(r\right)P_{0}\left[\frac{c\tau }{2}\right]\frac{A_{tel}}{r^{2}}\beta \left(\lambda ,r\right)e^{-2\int_{0}^{r}\alpha \left(\lambda ,r\right)dr}+P_{bg},$$

where $P_{0}$—peak laser pulse power;

*r*—the range at which the signal is received;

λ—laser wavelength;

$\eta_{all}$—overall efficiency of the LiDAR system;

*c*—speed of light;

τ—laser pulse duration;

$\eta_{q}\left(r\right)$—geometric factor (depends on the geometry of the LiDAR optical system, maximum value is 1);

$A_{tel}$—receiver aperture;

β(λ,r)—backscatter factor;

a(λ,r)—attenuation factor;

$P_{bq}$—background signal strength.

**Algorithm 1.** Finding the dependence of correct signal detection from “false alarms”**Input:** (1) the number of observations *n*;(2) observation vector $\bar{z}\left\{z_{1},z_{2},\ldots z_{n}\right\}$ *//* $\bar{z}\in G$ *(*$\bar{z}/G_{0}\in G_{0}$*;*$\bar{z}/G_{1}\in G_{1}$*);*$G_{0}+G_{1}=G;$ *The a priori probabilities of these n observations are unknown.***Output:** operating characteristic $P_{D}\left(P_{F}\right)$, i.e., dependence of correct signal detection from “false alarms” with given SNR values for $P_{F_{0}}=const=\varepsilon$1:H2=1 *// the hypothesis of the reception of a useful signal*2:H1=0 *// the hypothesis of noise reception*3:

$P_{D}\in p\left(\bar{z}/H2\right)\to H2$

4:

$P_{F}\in p\left(\bar{z}/H1\right)\to H1$

5:

$P_{F}=\iint_{G_{0}}p\left(\bar{z}/H1\right)d\bar{z}$

6:

$P_{D}=\iint_{G_{1}}p\left(\bar{z}/H2\right)d\bar{z}$

7:**if** $P_{F}+P_{D}=1$ **then**8:$P_{F_{H2}}=\alpha$ // *probability of error of type I (in which* $p(\bar{z}/H1)\to H2$*)*9:$P_{D_{H1}}=\beta$ // *probability of error of type II (in which* $p(\bar{z}/H2)\to H1$*)*10:**for** $\alpha =\varepsilon =P_{F_{0}}$ **find** β→min11:**for** $\int_{\lambda }^{\infty }p\left(\Lambda ∕H2\right)d\Lambda \leq \alpha$; $\alpha =p\left(\bar{z}/H2\right);\varepsilon =P_{F_{0}}=\int_{\lambda }^{\infty }p\left(\lambda ∕H2\right)$ **find***λ*12:

$\Lambda =\frac{p\left(z/H2\right)}{p\left(\bar{z}/H1\right)}\;\{\begin{array}{c} <\lambda \to H2\\ \leq \lambda \to H1 \end{array}$

13:    **for** q=SNR; $\sigma^{2}=\frac{\Sigma {\left(z_{i}-\bar{z}\right)}^{2}}{n}$ **find** $p\left(\bar{z}/H2\right)=\frac{z_{i}}{\sigma^{2}\left(1+q\right)}$ $e^{-\left(\frac{z_{i}^{e}}{2\sigma^{2}\left(1+q\right)}\right)}$ *// Rayleigh distribution*14:    **plot** $P_{D}\left(P_{F}\right)$ for $p_{F_{0}}=const=\varepsilon$; $q\epsilon \left\{q_{1},\;q_{2},\ldots q_{m}\right\}$15:
**end**


The backscatter and the attenuation factors can be generally represented as the sum of aerosol backscattering and molecular scattering factors respectively [74]:

(27) $$\beta \left(\lambda ,r\right)=\beta_{a}\left(\lambda ,r\right)+\beta_{m}\left(\lambda ,r\right)\;\alpha \left(\lambda ,r\right)=\alpha_{a}\left(\lambda ,r\right)+\alpha_{m}\left(\lambda ,r\right),$$

where *a* and *m* addends define the aerosol and molecular components for both backscatter *β* and attenuation *α* factors respectively.

The values of the variables in this equation depend on both the technical characteristics of the system itself, and the physical properties of the surrounding space and the characteristics of the probing pulse. Since the distance to the source of radiation increases, the useful signal scatters and the power of the incoming pulse at input of the optoelectronic system decreases significantly. In this case, it is quite difficult to extract the useful signal from the noise. However, there is another problem with the interference of scattered pulses, which can lead to two outcomes: increasing the pulse power (due to wave superposition) or complete loss of the pulses. These errors are unintentional and must also be taken into account when specifying the SNR threshold.

Let’s generate the signal pulse power statistics based on the equation below by simplifying the above equation:

(28) $$P\left(r\right)=\frac{\chi P_{0}A}{r^{2}}\;\frac{c\tau }{2}\beta \left(\lambda ,\;r\right)T_{1}\left(r\right)T_{2}\left(r\right)\frac{\varphi_{1}}{\varphi_{2}},$$

where χ—optical loss factor;

*r*—the distance to the object to be probed;

*P*_0—_peak laser pulse power;

*A*—receiver aperture;

*τ*—pulse duration;

*T*_1_(*r*)—the transparency of the atmosphere;

*T*_2_(*r*)—the transparency of the atmosphere for the return signal at its frequency;

$\varphi_{1}$—source frequency;

$\varphi_{2}$—return frequency.

**Figure 3:**
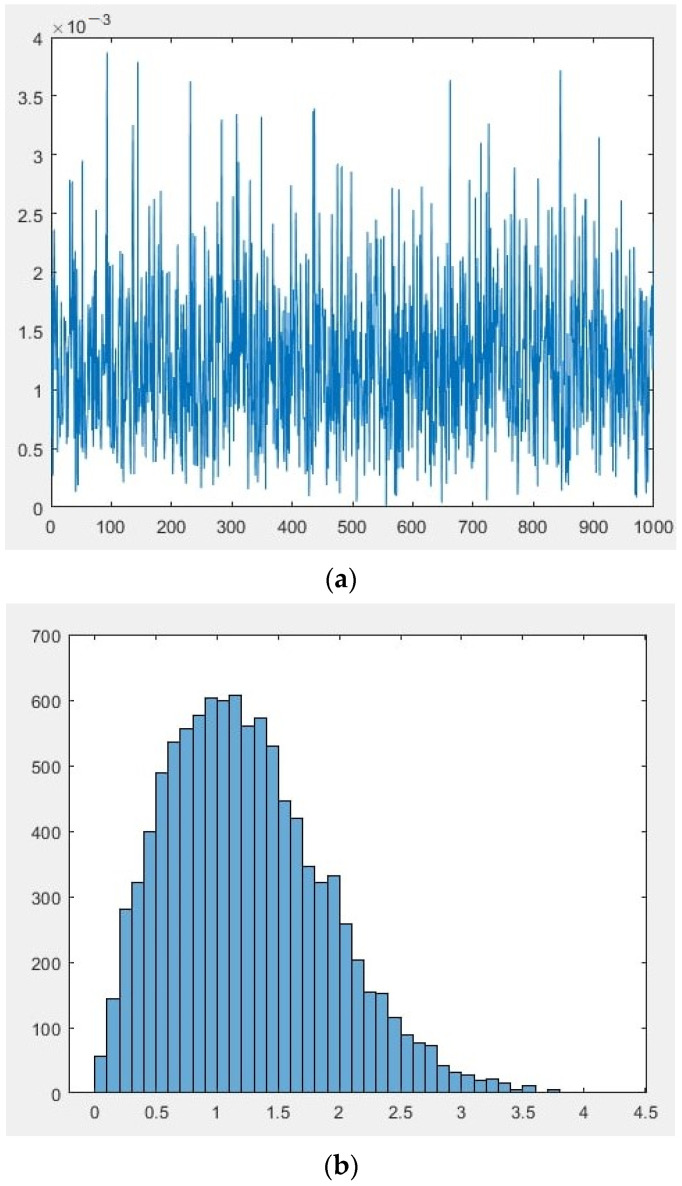
Generated power data for hypothesis H2: (**a**) power data for *N* = 1000; (**b**) histogram of observed data.

**Figure 4:**
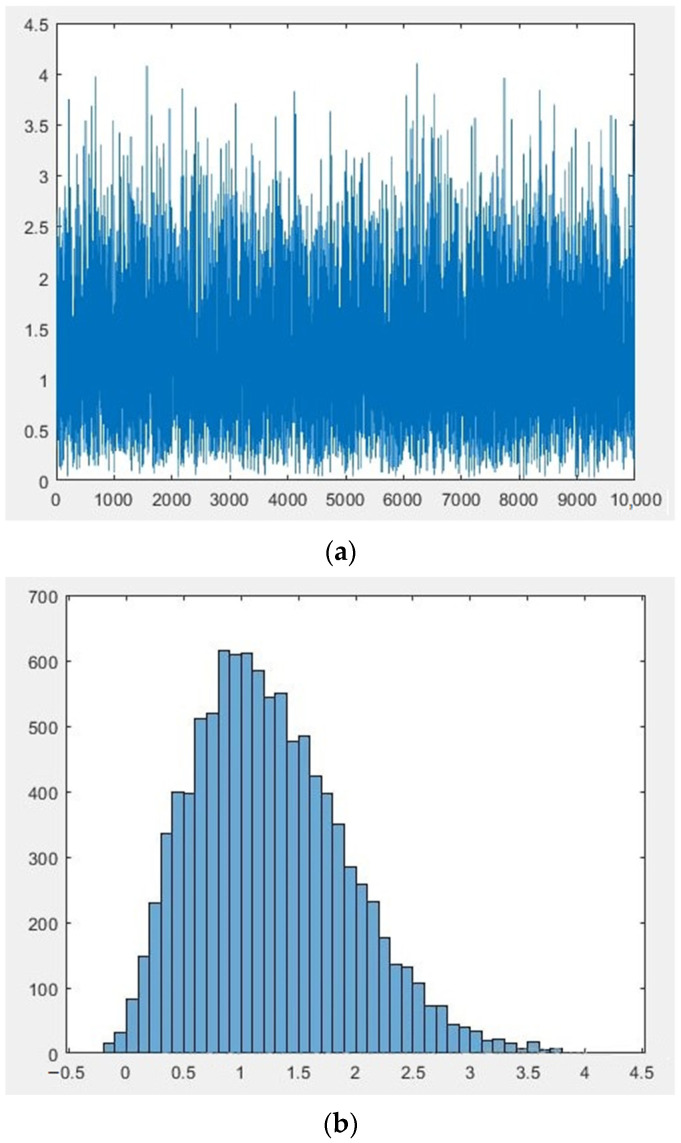
Generated power data for hypothesis H1 (with noise component to further isolate it): (**a**) power data for *N* = 1000 with noise component; (**b**) histogram of observed data.

Let’s present the generated data as graphs and histograms in Figure 3 and Figure 4 respectively.

The results of the measurement of the scalar component are a sample of *N* quantities: *p*_1_, *p*_2_, *p*_3_, …, *p_N_*, distributed according to the law similar to Rayleigh. According to both hypotheses H1 and H2, the measured variables pi are independent, equally distributed random variables with a zero mean. Under hypothesis H1, each of the variables has variance *σ*_01_, and under hypothesis H2, the variance is variance *σ*_12_.

### 6.5. Plotting the Graph of the Operating Characteristic Depending on the Selected SNR Value

For digital optoelectronic transmission systems there are two main methods for receiving optical signals (pulses) by the photodetectors: direct photodetection and hetero-dyne detection. When a pulse is received, a certain amount of current is generated. Therefore, a third factor, the noise variance in the photodetector, should be added to the threshold value of the SNR. Thus, the threshold value of the signal-to-noise ratio will be calculated as follows:

(29) $$\varpi =\frac{\sigma_{01}^{2}}{\sqrt{\sigma_{12}^{2}+\sigma_{nf}^{2}}}$$

The optimum threshold for the probability of occurrence of a “false alarm” is roughly equal to (30):

(30) $$P_{F}\approx 0.5\cdot \left[1-\Phi \left(\varpi /\sqrt{2}\right)\right],$$

where Φ(z)—error function calculated as:

(31) $$\Phi \left(z\right)=\frac{1}{\sqrt{2\pi }}\int_{0}^{z}exp\left(-\frac{x^{2}}{2}\right)dx.$$

**Figure 5:**
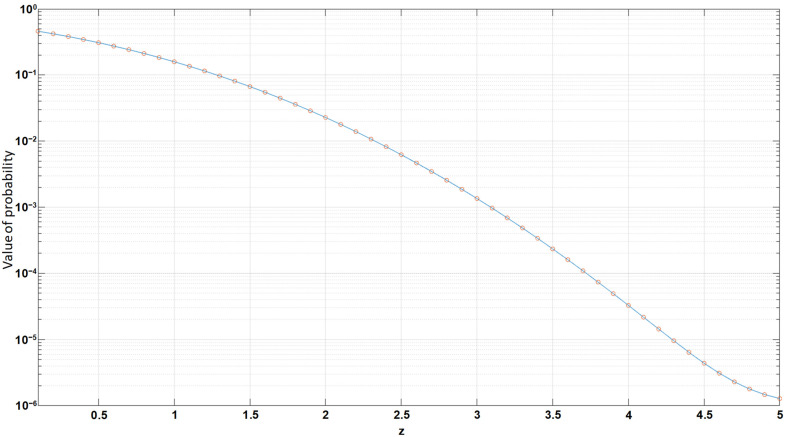
Dependence of error probability on signal to noise ratio threshold.

Now, it is possible to plot the error probability for different threshold values of the SNR based on (30). The error probability interval is similar to the one used in radiolocation and is defined in the range from 10^−6^ to 10^−3^ (Figure 5).

However, this dependence is built only for one value of signal-to-noise ratio (ϖ = 3). In order to construct a set of such dependencies and provide the methodical modeling error *δ_m_* = 10%, it is necessary to carry out at least $10^{8}$ (*M* ≥ $10^{8}$) total data computing iterations, with the number of observations not less than *N* = 100 for each. The required number of computing iterations can be calculated as:

(32) $$\frac{\sigma_{\varepsilon }}{P_{F0}}=\frac{\sqrt{1-P_{F_{0}}}}{\sqrt{MP_{F_{0}}}},$$

where $\frac{\sigma_{\varepsilon }}{P_{F_{0}}}=\delta m$—the relative error of the method, so if $P_{F0}=10^{-4}$ then *M* ≥ $10^{8}$.

**Figure 6:**
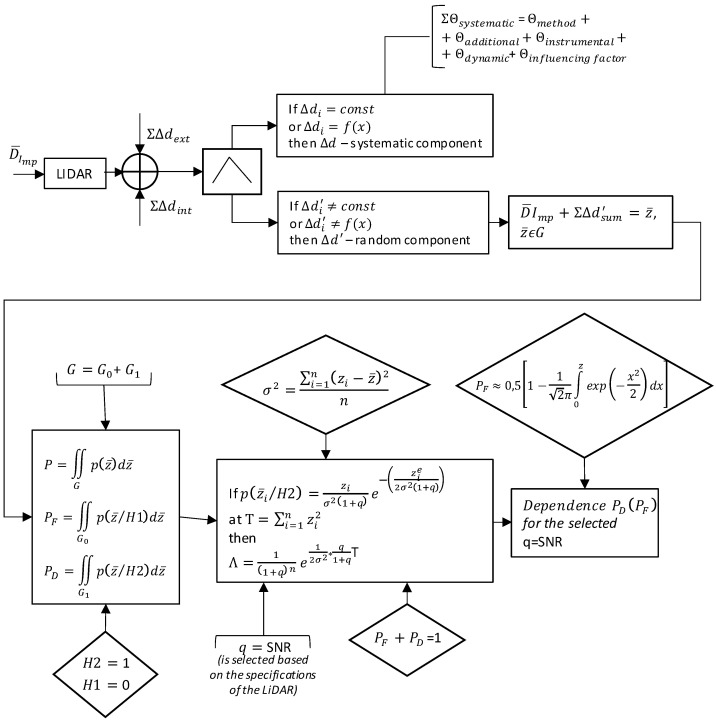
Flow chart of mathematical operations of the proposed approach.

The diagram presented in Figure 6 shows the stages needed to calculate the working characteristic, i.e., the dependency of the correct detection probability (with the Rayleigh distribution density function) on the probability of the “false alarm” event depending on the selected SNR value of the LiDAR.

### 6.6. An Example of Calculating the Received Optical Power of the Photodetectors Based on the Selected SNR Threshold

For quantitative studies, the challenge for developers is to strike a balance between the consideration of the instrumental parameters and the environmental factors. However, as mentioned earlier, the quantitative impact of each of the LiDAR subsystem parameter and environmental value on the final characteristics remain unclear. Therefore, it is necessary to study and evaluate the SNR at the output of the receiver subsystem, consisting of photodetector(s). This shall be the integral performance criterion.

LiDARs operate most commonly at 905 nm wavelength or close to it. Therefore, photodetectors with maximum sensitivity at the above wavelength have been considered.

Technical characteristics of Velodyne’s photodetectors have been considered for approbation purposes. This company uses the First Sensor series sensors in the production of its LiDAR systems. Photodetectors produced by Hamamatsu with similar characteristics have been considered for comparison. Table 1 shows the main technical characteristics of the photodetectors developed by First Sensor and Hamamatsu companies.

**Figure 7:**
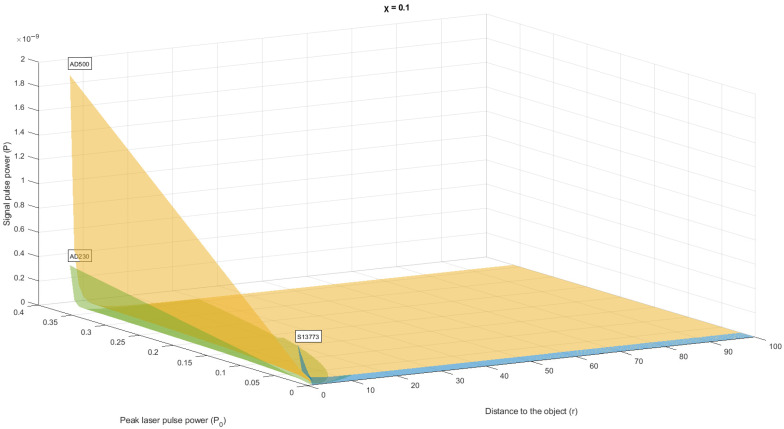
The dependence of the received optical power on the distance and pulse power of the LiDAR for three types of detectors (AD500, AD230 and S13773) in the media with optical loss factor $\chi_{1}$ = 0.1.

**Figure 8:**
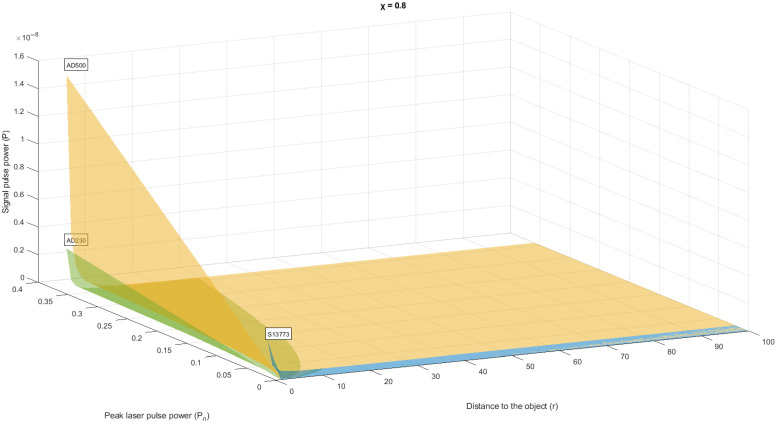
The dependence of the received optical power on the distance and pulse power of the LiDAR for three types of detectors (AD500, AD230 and S13773) in the media with optical loss factor $\chi_{2}$ = 0.8.

The ratio of the power of the noise component (interference overlay) to the power of the signal received is ≈ 1/10. Thus, the SNR will be within the range of 20–40 dB [61]. If the task is to ensure the value of the “false alarm” probability of 10^−3^, and the SNR is 30 dB, it is necessary to calculate the received optical power of the LiDAR’s receiver with the probability of correct signal detection to be not less than 0.95. Calculate the optical signal power based on the given technical characteristics in Table 1 by formula (28). Modelled data are shown in Figure 7 and Figure 8 for two different media with optical loss factor values $\chi_{1}$ = 0.1 and $\chi_{2}$ = 0.8.

The modelled data show the dependence of the received optical power from the distance to the object, taking into account the peak power of the transmitter of the LiDAR. In this case, graphical representation of the basic parameters of the receiver makes it easier to choose the required type of the photodetector for a particular LiDAR system.

Figure 7 and Figure 8 show that the vertices of optical power values obtained are different in height for different types of photodetectors. This indicates that, taken together, small changes in characteristics make a significant contribution to the overall component of the integral parameter, the optical power. The AD500-9 SMD photodetector has a big difference in power values, so it will be easier to separate the signal against the background noises using this photodetector.

The obtained calculation of the optical power supplements the approach described in the article. Given the fixed value of the “false alarm” and the resulting performance characteristic (in Figure 5), it is possible to choose the LiDAR system with necessary technical parameters.

### 6.7. Discussion

The dependence presented in the article may be one of the “operating characteristics” for the development and selection of optoelectronic measurement system. The operating parameters of the LiDAR’s photodetectors system are set as a point on the operating characteristic corresponding to a fixed threshold value. The type of this characteristic will be determined by the model of pulse power transfer in space, the level of noise and interference, the technical characteristics of LiDAR, the presence of random parameters in the detected signal, etc. The operating characteristic allows to determine the probabilities of correct detection and “false alarm” event depending on the selected optimum criterion. The article considers one operating characteristic, while a whole set of similar characteristics for different signal-to-noise threshold ratios are needed in practice in order to properly select the LiDAR’s operating mode. It is important to use several characteristics, since if a set is not created, the incorrect selection of the area of observation points under the same value of “false alarm” probability may lead to a significant reduction in the probability of correct detection by the LiDAR.

The calculation assumes that the useful signal is received together with an interference component (noise). The SNR at the input of the optoelectronic system (in terms of power) is in the range from 10^−6^ to 10^−4^. According to the statistical characteristics of signals, the output and input signals have been simulated for a given sample *N*. A histogram approximating the probability density function of output processes was repeated 1000 times for two hypotheses: when only noise is present at the input (hypothesis H1) and when a useful signal from a local source is received (hypothesis H2). The histograms approximating probability density function for noise and signal/noise reception allowed to calculate the probability of “false alarm” and probability of correct detection. The Neyman–Pearson criterion is used to select the detection rule that provides the minimum value of object missing (the maximum probability of correct detection) under the condition that the probability of false alarm does not exceed a given value.

## 7. Conclusions

In this work, a probabilistic approach to selecting the SNR threshold value based on the essence of the Neyman–Pearson criterion has been developed. It provides the minimum value of “ignoring” an object when scanning given that the “false alarm” does not exceed a given value.

The provided analysis shows that existing work on the impacts on the LiDARs and corresponding mitigation techniques are focused mainly on physical/hardware (e.g., using different types of modulation and optical frequency) and (less often) software-based (via the use of enhanced anomaly detection and data integrity verification systems, as well as improving the characteristics of filters for the points cloud data) approaches.

The analyzed works and researches do not offer methodological support for the design of LiDARs taking into account a priori estimation of the allowed SNR threshold and probability of detecting a reflected pulse, with no relation to preliminary estimates of probabilistic characteristics of LiDAR’s object detection.

It is worth noting that the proposed approach takes into account only the noise density function according to Rayleigh distribution. The mathematical description of the noise component of the photodetector considered the unintentional influence as an interference-induced error.

Future work implies the improvement of the proposed approach for other noise distributions, provided that the analytical expression for the established “false alarm” probability distribution is given.

## Figures and Tables

**Table 1:** Main electro-optical characteristics, t = 23 °C.

Symbol	Characteristic	Test Condition	Value	Unit
“First Sensor”				
AD230-9 SMD; AD230-9 TO				
	Active area		0.04	mm^2^
	Responsivity	M = 100; λ = 905 nm	52; 58; 60	A/W
	Quantum efficiency	λ: 750–905 nm	80	%
I_PEAK_	Peak DC current		0.25	mA
I_D_	Dark current	M = 100	0.5	nA
AD500-9 SMD				
	Active area		0.196	mm^2^
	Responsivity	M = 100; λ = 905 nm	52; 58; 60	A/W
	Quantum efficiency	λ: 750–905 nm	80	%
I_PEAK_	Peak DC current		0.25	mA
I_D_	Dark current	M = 100	0.8	nA
AD500-9-400M TO5				
	Active area		0.196	mm^2^
	Responsivity	M = 100; λ = 905 nm	52; 58; 60	A/W
	Quantum efficiency	λ: 750–910 nm	80	%
I_PEAK_	Peak DC current		0.63	mA
I_D_	Dark current	M = 100	0.8	nA
“Hamamatsu”				
Si PIN photodiodes S13773 and S15193				
	Active area		0.5	mm^2^
	Responsivity	M = 100	0.54; 0.64	A/W
	Quantum efficiency	λ: 785 nm; 830 nm	80	%
I_PEAK_	Peak DC current		0.1; 0.3	mA
I_D_	Dark current	M = 100	10	nA

## Data Availability

No new data were created or analyzed in this study. Data sharing is not applicable to this article.
